# *PDB_REDO*: constructive validation, more than just looking for errors

**DOI:** 10.1107/S0907444911054515

**Published:** 2012-03-16

**Authors:** Robbie P. Joosten, Krista Joosten, Garib N. Murshudov, Anastassis Perrakis

**Affiliations:** a Department of Biochemistry, Netherlands Cancer Institute, Plesmanlaan 121, 1066 CX Amsterdam, The Netherlands; b Structural Studies Division, MRC Laboratory of Molecular Biology, Cambridge CB2 0QH, England

**Keywords:** validation, refinement, model building, automation, PDB

## Abstract

Developments of the *PDB_REDO* procedure that combine re-refinement and rebuilding within a unique decision-making framework to improve structures in the PDB are presented. *PDB_REDO* uses a variety of existing and custom-built software modules to choose an optimal refinement protocol (*e.g.* anisotropic, isotropic or overall *B*-factor refinement, TLS model) and to optimize the geometry *versus* data-refinement weights. Next, it proceeds to rebuild side chains and peptide planes before a final optimization round. *PDB_REDO* works fully automatically without the need for intervention by a crystallographic expert. The pipeline was tested on 12 000 PDB entries and the great majority of the test cases improved both in terms of crystallographic criteria such as *R*_free_ and in terms of widely accepted geometric validation criteria. It is concluded that *PDB_REDO* is useful to update the otherwise ‘static’ structures in the PDB to modern crystallographic standards. The publically available *PDB_REDO* database provides better model statistics and contributes to better refinement and validation targets.

## Abstract 2

The decision-making algorithms and software used in *PDB_REDO* to re-refine and rebuild crystallographic protein structures in the PDB are presented and discussed.

## 1. Introduction

### 1.1. Validation

A crystallographic experiment and the ensuing process of phasing, model building and refinement (hopefully) culminates in a three-dimensional structure model that fits both the experimental X-ray data and our prior knowledge of macromolecular chemistry. Validation helps to guide this process and enables crystallographers to produce high-quality structure models suitable for biological interpretation. Validation software routines have been available since the early 1990s (Laskowski *et al.*, 1993 ▶; Hooft *et al.*, 1996 ▶; Davis *et al.*, 2004 ▶). These check many aspects of crystallographic (or other) structures, typically with a strong focus on macromolecules and specifically on proteins. Validation of nucleic acid structures is also available, for example in *MolProbity* (Chen *et al.*, 2010 ▶; Richardson *et al.*, 2008 ▶). Validation software for other chemical entities is available as well, *e.g. PDB-care* (Lütteke & von der Lieth, 2004 ▶) for carbohydrates and *WASP* (Nayal & Di Cera, 1996 ▶) for metal ions. Various validation tools are also available directly through graphical model-building software such as *O* (Jones *et al.*, 1991 ▶) and *Coot* (Emsley & Cowtan, 2004 ▶; Emsley *et al.* 2010 ▶).

While finalizing a crystallographic structure, validation can be used constructively to detect anomalies in the model and the crystallographer can either remove the anomaly or confirm that it is real, *i.e.* by assuring that there is sufficient (experimental) evidence for a model with this anomaly. This process has many complications. For example, large overall bond-length deviations typically arise from wrong or erroneous restraint settings; however, they can also be the result of errors in the experimental determination of unit-cell parameters and should not be resolved by tightening the restraints but rather by updating the unit-cell parameters.

Most validation tools focus on the identification of outliers: residues which are, based on statistical measures of our growing empirical knowledge of macromolecular structure, not similar to other residues in known structures. Focusing on outliers is sensible because these residues are most likely to be either interesting or wrongly modelled, but it takes away attention from the rest of the model. This becomes a problem when the absence of outliers is seen as a confirmation that the model is optimal. Side-chain rotamers are a good example: having no (or few) outliers does not mean all the rotamers are optimal with respect to the experimental data.

### 1.2. Validation after structure deposition

After a crystallographic structure has been finalized, deposited in the PDB and released, the atomic coordinates are set in stone. The Protein Data Bank (Bernstein *et al.*, 1977 ▶; Berman *et al.*, 2003 ▶) is a historical archive and does not change the atomic coordinates, although their annotation may be updated (Henrick *et al.*, 2008 ▶) to deal with the changing needs of depositors and users. The static nature of structure models in the PDB has many implications. The obvious consequence is that models that are several decades old often lack the accuracy and precision that modern crystallographic software offers, while they also lack the benefit of constructive validation which was either lacking or less ‘mature’ at the time these older models were constructed. This way, the users of the PDB often have access to models that are suboptimal by modern-day criteria. In addition, a less obvious consequence is that even when a user validates an existing PDB entry or uses ready-made validation reports as provided by, for example, the *PDBREPORT* data bank (Hooft *et al.*, 1996 ▶; Joosten, te Beek *et al.*, 2011 ▶), validation is no longer constructive: any anomalies found do not lead to an improved structure model because most PDB users do not have the crystallographic expertise to make the structure better before using it. In this context, validation may lead to the outright rejection of a structure model when an alternative with better validation results is available. This situation not only affects the older models in the PDB, which were accumulating at a slower pace (more crystallographic structures have entered the PDB in the last three years alone than in its first thirty years): a model submitted now will simply be an old model in a few years time, as crystallographic methods are still improving at an appreciable pace.

### 1.3. *PDB_REDO*

To keep PDB models up to date, we decided to apply some of the latest crystallographic methods to all PDB entries for which experimental X-ray data are available (Joosten, Salzemann *et al.*, 2009 ▶) and created the *PDB_REDO* software pipeline that takes atom coordinates and X-ray data from the PDB and re-refines the structure model in *REFMAC* (Murshudov *et al.*, 1997 ▶, 2011 ▶). A strong focus was placed on automation in order to deal with the tens of thousands of PDB entries, which meant dealing with the problems the PDB still had (at the time) with inconsistent annotation of coordinate and reflection files. The most important strength of automation is that it allowed us to optimize the weight between the X-­ray data and the geometric restraints, something that is a lot of work to do by hand even for a single structure model. In our re-refinement we consistently used TLS models for anisotropic atom movement (Schomaker & Trueblood, 1968 ▶; Winn *et al.*, 2001 ▶), which has only recently been made accessible to macromolecular crystallography, thus using the latest developments in refinement for all PDB entries. The resulting structure models showed an improvement in terms of *R*_free_ (Brünger, 1992 ▶), and model validation showed a substantial improvement in overall model-quality estimators such as the Ramachandran plot (Ramachandran *et al.*, 1963 ▶) *Z* score, which compares the combination of backbone torsion angles for each residue with a residue-type and secondary-structure specific distribution in high-quality protein structure models (Hooft *et al.*, 1997 ▶), and the number of atomic clashes or bumps. A notable result was that very recent PDB entries also improved, although in most cases the improvement was not as great or as common as in older PDB entries.

Making the improved structure models available to the PDB user community through the *PDB_REDO* data bank (Joosten & Vriend, 2007 ▶) was an initial step towards a constructive form of structure validation of deposited structure models. However, it was clear that local fitting errors and other problems in structure models could not be resolved by the approach we used (Joosten, Womack *et al.*, 2009 ▶). A much more comprehensive approach that incorporates real-space model rebuilding was needed.

### 1.4. Real-space rebuilding

Algorithms for real-space fitting and rebuilding parts of the structure model have existed for decades (Diamond, 1971 ▶; Jones, 1978 ▶). The problem lies in deciding *how* they should be applied and *where* in the structure. The typical approach has been to manually update the structure model using molecular graphics, with a validation report to hand. The program *OOPS* (Kleywegt & Jones, 1996 ▶) provided a significant speed-up of this process by automating the *where* part of the problem: it used validation results from *O* (Jones *et al.*, 1991 ▶) and *WHAT_CHECK* (Hooft *et al.*, 1996 ▶) and turned them into a ‘macro’ for *O* that takes a user through the flagged parts of the structure model automatically. Similar implementations are available to interface *MolProbity* (Chen *et al.*, 2010 ▶) with *Coot* (Emsley *et al.*, 2010 ▶). Solving the *how* part of the problem still remains a challenge in structure validation: finding an anomaly is often easier than understanding what caused it.

We recently described two programs (*pepflip* and *SideAide*) that rebuild structure models using a strategy that incorporates both the *where* and the *how* in a single decision-making framework (Joosten, Joosten *et al.*, 2011 ▶). Instead of improving crystallographic structures just by rebuilding the parts of the model flagged by validation routines, the main chain and side chain of every residue is rebuilt and validated; if rebuilding leads to an improved fit to the crystallographic maps, then the model is updated with the new conformation. Instead of specifically looking for errors, this approach focuses on improvable features of the structure model and therefore has much greater coverage of the structure model than validation-based methods. The algorithms are fully automated and not particularly computationally intensive, enabling us to incorporate them in the *PDB_REDO* pipeline.

The main challenge in automation lies in decision making. Decisions, and the priority they are given, are often taken based on statistic measures, but also on personal preference, experience or gut feeling, or even on dogmatic principles. Many decisions are taken without even realising it, *e.g.* by applying default values to many parameters. Fully automated software pipelines need to formalize all these decisions to a closed set of rules. Here, we discuss *PDB_REDO* as a decision-making framework, showing the choices we face and how we resolved them, and also present an extensive test on 12 000 PDB entries.

## 2. Methods

The different model-quality metrics used in the decision-making process of model optimization are described in Table 1 ▶. The software programs used in the procedure are discussed in the text and summarized in Table 2 ▶.

### 2.1. The *PDB_REDO* pipeline

The *PDB_REDO* software pipeline uses the PDB file with the deposited crystallographic structure coordinates and the associated reflection file containing the X-ray diffraction data.

#### 2.1.1. Preparation of the diffraction data (CIF file)

The reflection file is standardized with *Cif2cif* (Joosten, Salzemann *et al.*, 2009 ▶), which writes out Miller indices and amplitudes (or intensities if amplitudes are not given) for each reflection. σ values and *R*_free_-set flags are written out if available. Only the first data set in the reflection file is used. Some basic sanity tests are performed as follows.

(i) Reflections with negative values are rejected if they are marked as amplitudes, but are kept if they are intensities.
(ii) If σ values are given the set is tested for information content. If all values are the same, the σ values cannot be used for scaling purposes in refinement. Individual σ values of 0.0 are reset to the highest σ value in the data set.
(iii) The *R*_free_ set is validated, as discussed below (§[Sec sec2.2.1]2.2.1).

The standardized data set is then completed and converted to an MTZ file using tools from the *CCP*4 suite (Winn *et al.*, 2011 ▶). Intensities are converted to amplitudes using *CTRUNCATE*, which deals with negative intensities (French & Wilson, 1978 ▶). The *B*_Wilson_ is calculated by *SFCHECK* (Vaguine *et al.*, 1999 ▶). *SFCHECK* is also used to check the data completeness and to check for the presence of twinning.

#### 2.1.2. Preparation of the coordinates (PDB file)

The PDB file is parsed with *Extractor* to extract cell dimensions, space group, waters and special residues that are involved in chemical interactions (as marked in LINK records in the PDB header). Parameters about refinement are also extracted at this stage: the *R* factor and the *R*_free_ from the PDB header (*R*_head_, *R*_free,head_), TLS-group selections and tensors, and the type of solvent model. The following records are removed from the PDB file by the program *Stripper*, mostly to ensure proper restraint generation for refinement: explicit H atoms, atoms with occupancy < 0.01, superfluous O atoms in carbohydrates, if detected by *PDB-care*, unknown ligands (UNLs), side-chain atoms beyond C^β^ for unknown residues (UNKs), inter-symmetry LINK and SSBOND records, fields containing atomic distances in LINK records, unknown atoms (UNXs) and any other atoms of element X since no scattering factors can be assigned.

#### 2.1.3. Calculation of baseline values for model quality

The original structure is validated using *WHAT_CHECK*. *REFMAC* is then used (without any refinement) to calculate *R*_calc_ and *R*_free,calc_ in five steps.

(i) The ‘TLS ambiguity’ is resolved first: are the *B* factors of the model ‘total’ or ‘residual’? To decide, a run with and without the TLS model (if available) is attempted and the approach that gives the lowest *R*_calc_ is chosen.
(ii) If the difference between the calculated *R* factor (*R*_calc_) and that extracted from the header of PDB file (*R*_head_) is more than 5% (*R*_calc_ − *R*_head_ > 5%) and a twin fraction of >5% was detected by *SFCHECK*, then *REFMAC* is run with twinning.
(iii) If the above difference persists, rigid-body refinement is tried.
(iv) If a difference of above 5% still persists, then five cycles of TLS refinement are tried (only if TLS tensors were extracted from the PDB file header) in an attempt to deal with possible corruption of the TLS tensors.
(v) If after these steps the difference between the reported and the calculated metrics is more than 10%, it is decided that something is inherently wrong with this PDB entry and the *PDB_REDO* pipelines stops prematurely.

New restraint files for compounds not yet in the *REFMAC* dictionary (Vagin *et al.*, 2004 ▶) are generated automatically. The final values of *R*_calc_ and *R*_free,calc_ plus those of *R*_ratio_, *R*_free,unb,calc_, *Z*(*R*_free,calc_), r.m.s.*Z*(bond)_calc_ and r.m.s.*Z*(angle)_calc_ are used as baseline values for further refinement.

#### 2.1.4. Re-refinement

To get the most out of refinement in *REFMAC*, many parameters should be optimized. In the original *PDB_REDO* pipeline we only optimized the geometric restraint weight. Despite the presence of a robust and reliable option to automatically weight X-ray data and geometry in newer versions of *REFMAC*, we find that our search method can have advantages. We use the geometry-weight optimization and also systematically explore the usage of many other refinement strategies, as follows.

(i) If *SFCHECK* finds a twin fraction of >5%, twinning is evaluated in *REFMAC*. If *REFMAC* finds twin operators with *R*_merge_ < 44% and twin fraction >7%, refinement is performed with twin target functions.
(ii) Four different *B*-factor models are evaluated: anistropic *B* factors, isotropic *B* factors with TLS, isotropic *B* factors without TLS and one overall *B* factor with TLS. The selection of the optimal algorithm is discussed below.
  (1) If it is decided that anisotropic *B* factors cannot be used, the possibility of TLS refinement is tested by resetting all atomic *B* factors to the *B*_Wilson_ (or the average *B* factor at resolutions 4 Å or worse) and calculating *R*_TLS_ and *R*_free,TLS_, followed by TLS refinement alone. Multiple TLS models are tested whenever possible: a simple model with one TLS group per chain, the TLS model extracted from the PDB header and any additional user-provided TLS models (for instance, from *TLSMD*; Painter & Merritt, 2006 ▶). The selection algorithm for the best TLS model is also discussed in §[Sec sec3]3. If TLS refinement decreases *R*_free_ with respect to *R*_free,TLS_, the output TLS model will be used in further refinements as is.
  (2) If individual *B* factors are used, the weight of the *B*-­factor restraints (which keep neighbouring *B* factors similar) is optimized by performing a grid search of up to seven different values. For each weight a short refinement is performed with automatic geometric restraint weighting. The selection algorithm for the best weight is discussed in §2.2.4.

After all these choices have been made, the actual re-refinement is performed. A riding hydrogen model is always used to optimally benefit from van der Waals restraints. Local NCS restraints (Murshudov *et al.*, 2011 ▶) are used when applicable, regardless of the data resolution. In this re-refinement, up to seven different geometric restraint weights are used, meaning that a final model will be selected from a number of candidates; the selection algorithm is also discussed in §2.2.4.

Our previous *PDB_REDO* results showed that re-refinement could benefit from making the search space for the geometric restraint weight resolution-dependent. We have performed this by assigning each input model to one of six different categories (Table 3 ▶) based on the resolution of the data and the number of X-ray reflections per atom. The latter is important because at a given data resolution the number of reflections per atom varies greatly depending on the solvent content: for example, at 3.0 Å resolution values of between 1.9 and 7.8 reflections per atom are observed. In addition to assigning the geometric restraint-weight search space, the ‘resolution’ categories are also used for determining the *B*-­factor restraint-weight search space and the application of ‘jelly-body’ restraints (Murshudov *et al.*, 2011 ▶), which we use to stabilize the re-refinement of structures belonging to the two lowest resolution categories (‘vlow’ and ‘xlow’).

#### 2.1.5. Rebuilding

The re-refinement results in a structure model with new (difference) electron-density maps. These maps are used to rebuild the structure model in four steps.

(i) The program *Centrifuge* removes all waters with low weighted mean density fit to the 2*mF*_o_ − *DF*_c_ map [fit(ρ) < 0.37]. This resolution-independent cutoff value used within *PDB_REDO* was optimized to minimize the false-positive rate, *i.e.* to ensure that waters were not deleted where they should be kept; however, water deletion and eventual addition remains a major area with need for future development.
(ii) The program *pepflip* (Joosten, Joosten *et al.*, 2011 ▶) is used to flip the orientation of peptide planes in the model if this improves the fit of the peptide with, in order of importance, the *mF*_o_ − *DF*_c_ map at the position of the oxygen, the 2*mF*_o_ − *DF*_c_ map of the whole peptide and a combination of the two fits and the geometry, while maintaining or improving the backbone torsion angles with respect to the Ramachandran plot (Ramachandran *et al.*, 1963 ▶) of the two residues involved.
(iii) The side chains are rebuilt in rotameric conformations, followed by refinement in real space, by the program *SideAide* (Joosten, Joosten *et al.*, 2011 ▶) if this improves the fit to the 2*mF*_o_ − *DF*_c_ map. Missing side chains are added. Water molecules that are erroneously built at side-chain positions are removed in the process.
(iv) The model is validated with *WHAT_CHECK* and the results are used in a separate *SideAide* run to flip His, Asn and Gln side chains to improve hydrogen bonding, to flip Asp, Glu, Phe and Tyr side chains to standardize the χ_2_ angle (χ_3_ for Glu), unswap mixed-up N^∊^ atoms in Arg to standardize the geometry and finally to fix (administrative) chirality errors in the C^β^ atom of Thr, Ile and Val and the C^γ^ atom of Leu. This second side-chain rebuilding run is needed to fix side chains that were not rebuilt in the previous step.

Only structures in the ‘atomic’, ‘high’, ‘medium’ and ‘low’ categories are included in rebuilding steps (ii), (iii) and (iv), since the maps are generally not clear enough for un­supervised rebuilding for the ‘vlow’ and ‘xlow’ categories.

The rebuilt model is refined in *REFMAC* one last time. The TLS model (if used) is updated, followed by restrained refinement with the previously established refinement parameters but with three different geometric restraint weights: the optimal weight from the re-refinement, a slightly tighter restraint weight and a slightly looser one. The final model is selected using the same selection algorithm as used in the re-refinement.

#### 2.1.6. Final output

**Figure 1:**
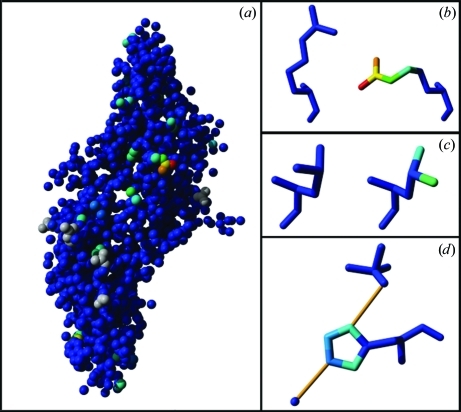
*YASARA* scene showing the changes made to PDB entry 2ask (Silvian *et al.*, 2006 ▶) by *PDB_REDO*. The atoms are coloured by atomic shift, with warmer colours marking larger shifts. (*a*) Overview of the structure model with atoms as spheres. Grey atoms were newly built by *SideAide*. (*b*) The residue with the greatest atomic shift (Arg*A*85) before (left) and after *PDB_REDO*. The side chain is moved to a completely different rotamer. (*c*) The rotamer change in Leu112 has led to a large displacement of the C^δ^ atoms, whereas the C^γ^ atom has hardly moved. (*d*) His*A*32 with typical colouring for a side-chain flip. In the new conformation the side chain makes hydrogen bonds (thin orange rods) to sulfate *A*504 and water *A*508.

The final model is validated with *WHAT_CHECK*. The validation scores and the *R* and *R*_free_ values are combined to give a web page for the *PDB_REDO* data bank and an unformatted file that can be used for data mining. Three-dimensional scenes are created that show the model atoms coloured by atomic movement, with warmer colours marking increasing atomic shifts with respect to the original structure model (Fig. 1 ▶), and the model atoms coloured by TLS group. The scenes can be visualized in the free viewer version of *YASARA* (Krieger *et al.*, 2002 ▶). A plug-in for *Coot* (Emsley *et al.*, 2010 ▶) is available to download and visualize *PDB_REDO* optimized structure models and their electron-density maps.

### 2.2. Decision-making algorithms in *PDB_REDO*

To be able to apply the *PDB_REDO* pipeline to the entire PDB, it was necessary to create decision-making algorithms that can deal with the many choices available when optimizing structure models without supervision or user input. Here, we show the result of our developments in the five main decision-making algorithms.

#### 2.2.1. Using the *R*_free_ set

The introduction of the *R*_free_ metric (Brünger, 1992 ▶) was an important step in macromolecular crystallography as it helped to detect overfitting and overrefinement of the model. However, until the mid-1990s many structures were refined without using *R*_free_. Properly dealing with *R*_free_ sets is therefore essential for structure-model optimization; *PDB_REDO* deals with *R*_free_ sets using the following procedure.

(i) The size of the *R*_free_ set is checked. If it is greater than the work set the sets are swapped. The *R*_free_ set is rejected if it contains more than 25% of all reflections or fewer than 500 reflections.
(ii) If an *R*_free_ set is not available, it is created using of 5% of the reflections, but in this case *R*_free,calc_ is treated as ‘biased’. If this set consists of less than 1000 reflections the percentage of reflections for the free set is increased to a maximum of 10%.
(iii) Test to see if the *R*_free_ set is really ‘free’, *i.e.* attempt to make sure that it was not previously used in refinement. The *R*_free,calc_ is treated as ‘biased’ if the following apply.
  (1) A new *R*_free_ set was created in the step above.
  (2) *R*_free_ is less than *R* (*R*_free,calc_ <* R*_calc_).
  (3) *R*_free,calc_ is much lower than expected with respect to *R*_free,calc,unb_ [*Z*(*R*_free,calc_) > 10.0].
  (4) The difference between *R*_free,calc_ and *R*_calc_ is much smaller than expected [*R*_free,calc_ − *R*_calc_ < 0.33 × (*R*_free,head_ − *R*_head_)].
(iv) If *R*_free,calc_ is biased it is not used as baseline value for the structure optimization. *R*_free,calc,unb_ is used instead. At the same time the refinement protocol is adapted by increasing the number of refinement cycles and by resetting the atomic *B* factors to *B*_Wilson_. When the refinement converges the new *R*_free_ values are considered to be ‘free’ again.

#### 2.2.2. Selecting a *B*-factor model

The atomic displacement factors, commonly referred to as *B* factors, can be parameterized to represent various levels of detail: anisotropic *B* factors require nine parameters per atom, isotropic *B* factors four and a single *B* factor for all atoms only three. In *PDB_REDO* we assign the type of *B*-factor model based on the number of X-ray reflections per atom (RPA) using the following successive criteria.

(i) If the atomic parameters including anisotropic *B* factors are twofold overdetermined, use anisotropic *B* factors (RPA > 18).
(ii) If there are still 50% more reflections than parameters including anisotropic *B* factors (18 > RPA > 13.5) the atomic *B* factors are set to *B*_Wilson_ and two refinements using isotropic and anisotropic *B* factors are run using automatic geometric restraint weighting and default *B*-factor restraints. The program *bselect* (see below) is then used to pick the best *B*-­factor model.
(iii) If there are more than three reflections per atom (13.5 > RPA > 3) isotropic *B* factors are used.
(iv) If there are fewer than three reflections per atom (RPA < 3) the TLS model is optimized first. The TLS model and automatic geometric restraint weighting are used for a refinement with isotropic *B* factors and tight *B*-factor restraints and a refinement with an overall *B* factor only; *bselect* is then used to pick the best *B*-factor model.
(v) If TLS cannot be used (*e.g.* because it is unstable in refinement) then isotropic *B* factors are used.

Making the *B*-factor model more detailed adds a large number of extra parameters to the structure model. This typically causes a drop in the *R* factor but also in *R*_free_ (Bacchi *et al.*, 1996 ▶). As a result, a drop in *R*_free_ cannot be used to decide whether or not a more detailed *B*-factor model is acceptable. The Hamilton test (Hamilton, 1965 ▶; Bacchi *et al.*, 1996 ▶) provides a way to test the significance of a perceived model improvement resulting from adding more parameters to a model by looking at the ratio of *R*_w_ or *R*_free,w_ values for the simple and the more complex model. The problem with this method is that the degrees of freedom of the simple and the complex model must be known, which requires knowledge of the number of experimental data points and model parameters and the effective number of model restraints. The latter number can be described as the absolute number of restraints multiplied by a weight *w*. The value of *w* is not known, but a recent implementation of the Hamilton test (Merritt, 2012 ▶) circumvents this issue by establishing a range for *w*_1_ for the basic restraints in both models and *w*_2_ for the extra restraints in the complex model and then checking all the possible values. The program *bselect* uses this method and other criteria to decide between two *B*-factor models in successive steps.

(i) If the weighted value for the more complex model is higher than that of the simpler model (*R*_free,w,complex_ > *R*_free,w,simple_) the simpler model is used.
(ii) All possible combinations of *w*_1_ and *w*_2_ are used for Hamilton tests. The percentage of tests that gave an acceptable result (where ‘acceptable’ means that the more complex model is appropriate) is then calculated. If less than 30% of the Hamilton tests were acceptable, the simpler model is used. If more than 95% of the Hamilton tests were acceptable, the more complex model is used.
(iii) If the step above is inconclusive (acceptable tests between 30 and 95%), the complex model is examined by looking for signs of overrefinement and the simpler model is used if the *Z* score for *R*_free_ is too low [*Z*(*R*_free_)_complex_ < −3.0]. If *Z*(*R*_free_)_complex_ cannot be calculated reliably (when the *R*_ratio_ calculation fails) we check the difference between the *R*_free_ and *R* of the complex model: if this is larger than a cutoff value (*R*_free,complex_ − *R*_complex_ > *cutoff*, where *cutoff* is 4% for anisotropic *B* factors and 6% for isotropic *B* factors) then the simpler model is used. Finally, if the difference between the *R*_free_ and the *R* factor for the complex model is more than two times the difference for the simpler model [(*R*_free,_ _complex_ − *R*_complex_) > 2.0 × (*R*_free,_ _simple_ − *R*_simple_)] then the simpler model is used. Otherwise, we consider that there are no signs of overrefinement and the more complex model is used.

#### 2.2.3. Selecting the number of refinement cycles

A large number of *REFMAC* refinement runs are performed to try different parameters; thus, the number of internal refinement cycles becomes an important contributor to the time needed to optimize a model. Based on our experience with *REFMAC*, we use a set of empirical rules to assign the number of refinement cycles.

(i) For rigid-body refinement, we use ten cycles when attempting to reproduce the *R* factor reported in the PDB header, *R*_head_ (see §2.2.2). Rigid-body refinement is also used for ‘legacy’ structure models predating 1990; in this case, 15 cycles are used since older structure models may be further away from convergence.
(ii) For TLS refinement, we use five cycles for reproducing *R*_head_ (see §2.2.2), ten cycles for optimizing the TLS model (15 cycles for ‘legacy’ models) and five cycles in the final model refinement.
(iii) During re-refinement, 20 cycles are used by default. If no TLS models are used, five additional cycles are introduced. In the cases where a new *R*_free_ was created 30 cycles are used, and finally when using anisotropic *B *factors 40 cycles are used since anisotropic refinement takes longer to converge. For ‘legacy’ models, 50 cycles are used by default and 60 cycles are used when using anisotropic *B* factors.
(iv) For choosing the *B*-factor model and weights, we use the same number of cycles as in re-refinement when deciding between individual isotropic *B* factors or one constrained *B* factor. For deciding between isotropic and anisotropic *B* factors 50 cycles are used. Finally, ten cycles are used for optimizing the *B*-factor restraint weight with TLS models and 15 cycles when TLS models are not used.

#### 2.2.4. Selection of the optimal model from a set of refinement results

The TLS-model optimization, *B*-factor restraint-weight optimization, re-refinement and final refinement require the selection of the best model from a set of refinement results. The program *picker* selects the model with the best fit to the experimental data while minimizing the risk of overrefinement. To do this, the quality of the starting structure is also taken into account. *Picker* uses the following procedure.

(i) Firstly, we establish *R*_co_ and *R*_free,co_ as cutoff values depending on the source of the models in question. *R*_TLS_ and *R*_free,TLS_ are used if the models originate from TLS-model optimization refinement; *R*_calc_ and *R*_free,calc_ are used in all other cases. If *R*_free,calc_ has been considered to be ‘biased’ then *R*_free,unb,calc_ is used instead of *R*_free,calc_.
(ii) Similarly, r.m.s.*Z*(bond)_co_ and r.m.s.*Z*(angle)_co_ are established as geometry cutoff values. The default cutoff values are 1.0, but the values are increased to r.m.s.*Z*(bond)_calc_ and r.m.s.*Z*(angle)_calc_, respectively, if these were greater than 1.0.
(iii) For each refinement condition *i*, *Z*(*R*_free_)_*i*_ and σ(*R*_free_)_*i*_ are calculated. From these, a maximum value for *R*_free_ is calculated [*R*_free,max,*i*_ = *R*_*i*_ × *R*_ratio_ + 3.5 × σ(*R*_free_)_*i*_]. *R*_free,max,*i*_ is then set to the lower of *R*_free,max,*i*_ and (*R*_*i*_ + 6%) to make sure that the difference between *R* and *R*_free_ is not too large. Finally, *R*_free,max,*i*_ is set to the higher of *R*_free,max,*i*_ and (*R*_free,co_/*R*_co_ × *R*_*i*_) to deal with structures that had a high initial *R*_free_/*R* ratio.
(iv) After the cutoff values have been established, any model is rejected if a model metric exceeds the preset limits [r.m.s.*Z*(bond)_*i*_ > r.m.s.*Z*(bond)_co_, r.m.s.*Z*(angle)_*i*_ > r.m.s.*Z*(angle)_co_, *R*_free,*i*_ > *R*_free,max,*i*_, *R*_free,*i*_ > *R*_free,co_). For structures in the ‘vlow’ and ‘xlow’ categories the difference between *R*_free_ and the *R* factor is also taken into account for model rejection [(*R*_free,*i*_ − *R*_*i*_) > 2.0 × (*R*_free,co_ − *R*_co_)].
(v) Finally, the optimal refinement is selected from the remaining conditions. The models with the lowest free likelihood (LL_free_) and the lowest *R*_free_ are selected. If these two models are different, that with the highest *Z*(*R*_free_) is finally selected.

The algorithm in *picker* rejects all refinements if none pass the established criteria and the *PDB_REDO* pipeline adapts accordingly. Depending on the set of refinements evaluated by *picker*, TLS might not be used further in refinement or the *B*-­factor restraint weight can be set to the *REFMAC* default. If the evaluation after re-refinement does not produce a better model, the original structure is used in the subsequent rebuilding steps and the final refinement is performed with auto weighting for the geometric restraints.

#### 2.2.5. Selection of atoms for exclusion from rebuilding

The first three steps of rebuilding are meant to be comprehensive, so by default all residues are considered. Negative selection is applied to ‘special’ residues for which automated unsupervised rebuilding is too risky or not needed.

(i) *Centrifuge* ignores all waters involved in LINKs (see §2.1.2).
(ii) *Pepflip* ignores all residues in which the backbone N or the backbone O atom is involved in a LINK. Residues in the middle of secondary-structure elements, as designated by *DSSP* (Kabsch & Sander, 1983 ▶), are very unlikely candidates for flips. They are ignored as well to speed up peptide flipping.
(iii) *SideAide* ignores all side chains involved in LINK records and all side chains with multiple conformations.

The final rebuilding step uses positive selection of candidates based on a *WHAT_CHECK* validation report. However, the negative selectors above still apply.

### 2.3. Evaluation data set

We attempted to optimize all of the PDB entries with deposited X-­ray diffraction data (>53 000) for inclusion in the *PDB_REDO* data bank. Fewer than 900 PDB entries (<2%) cannot be used at the moment owing to various problems. The reasons for these missing entries are listed in the *WHY_NOT* data-bank annotation server (Joosten, te Beek *et al.*, 2011 ▶). Some of the reasons for the exclusion of structures from the *PDB_REDO* data bank are as follows.

(i) The atomic coordinates do not describe the entire asymmetric unit. This is mostly the case for PDB entries split over multiple files owing to limitations in the PDB format.
(ii) No *R* factor is given in the PDB header and it cannot be recovered from the literature. This is mostly a problem with unrefined low-resolution assemblies.
(iii) The *R* factor cannot be reproduced to within ten percentage points of the reported value. This can be caused by limitations in our current methods, but also by partially missing data or by deposition of the wrong experimental data.
(iv) The structure was determined by ‘other’ diffraction methods such as neutron, fibre or powder diffraction.
(v) The model contains only C^α^ atoms.

Here, we discuss the results obtained based on a random subset of the PDB consisting of 12 000 structure models deposited between 1995 and 2010.

## 3. Results and discussion

### 3.1. High-throughput testing

The new *PDB_REDO* pipeline was tested with our evaluation data set of 12 000 PDB entries. Table 4 ▶ shows a summary of structure-quality metrics for the data set in the original PDB entry, the re-refined structure model and the final re-refined and rebuilt structure model. On average the improvement in *R*_free_ was 1.8%, corresponding to a significant relative improvement of 6.4σ(*R*_free_). A total of over 70 000 unsatisfied hydrogen-bond donors or acceptors and more than 310 000 atomic bumps were removed. Over 200 000 erroneous water molecules were removed and 57 000 previously missing side chains were built in total. On average, only a single peptide flip was needed in every second model (still accounting for about 7000 wrongly modelled peptides in total). The least common fix of those attempted was explicit chirality fixes of threonine, valine and isoleucine C^β^ atoms and leucine C^γ^ atoms, which were applied only 12 times in the entire data set.

**Figure 2:**
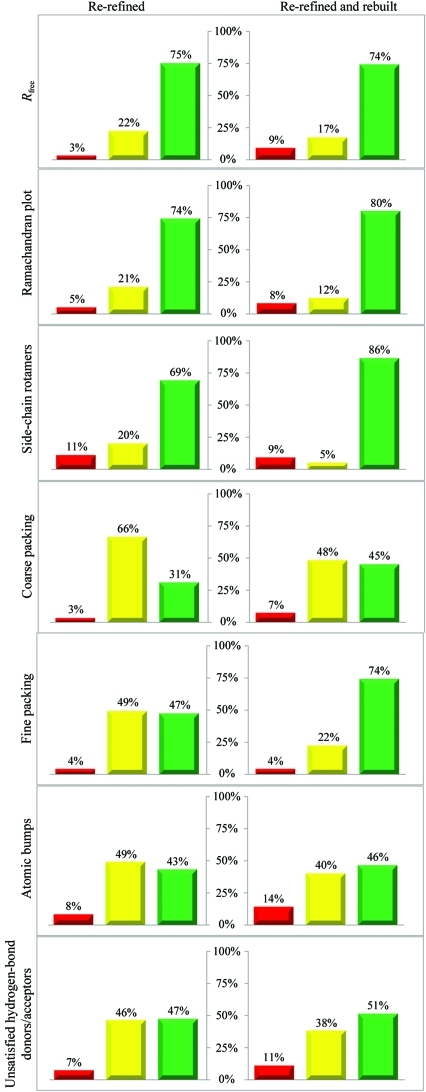
Traffic-light diagrams of change in structure model-quality metrics after re-refinement (left column) and after full model optimization (re-refined and rebuilt; right column) for 12 000 structure models. Green bars represent improved structure models and red bars deteriorated models. Models are considered to be the same (yellow bars) if |Δ*R*_free_| ≤ 2σ(*R*_free_), |Δ*Z* score| ≤ 0.1 (for Ramachandran plot, rotamers, coarse and fine packing), |Δ(No. of bumps)| ≤ 10 or |Δ(No. of unsatisfied hydrogen-bond donors/acceptors)| ≤ 2.

**Figure 3:**
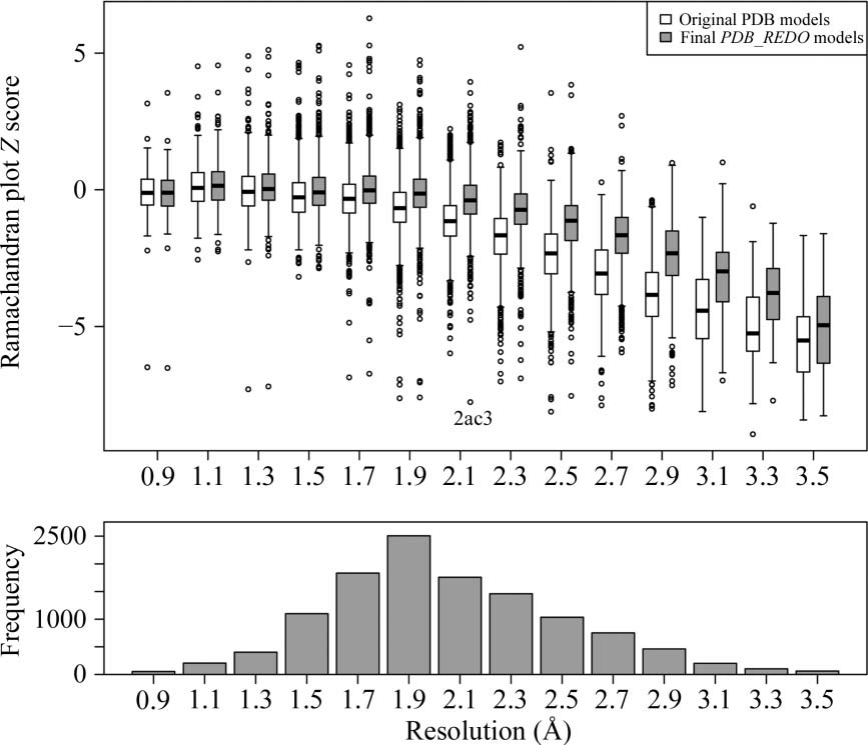
Box-and-whisker plot of the Ramachandran plot *Z* score (higher is better) of the original PDB entries (white) and the fully optimized *PDB_REDO* models (grey) in 0.2 Å resolution bins; the size of each bin is given in the bar chart. One severe outlier, PDB entry 2ac3 (Jauch *et al.*, 2005 ▶), was caused by a TLS-related bug in *PDB_REDO*. With the latest version of *PDB_REDO*, the final *Z* score is −1.1.

To obtain a better perspective on the performance of *PDB_REDO* on individual PDB entries, we made ‘traffic-light’ diagrams for the structure-quality metrics (Fig. 2 ▶). Each diagram shows the percentage of structure models that became better, stayed the same or became worse after re-refinement and rebuilding, according to different metrics. Depending on the metric used, 31–75% of the models improved on re-refinement and 45–86% of the models improved on full optimization including rebuilding. The greatest improvements are found in *R*_free_ and in the side-chain rotamer and Ramachandran plot *Z* scores (Hooft *et al.*, 1997 ▶). The first two are explicitly optimized and such a result would be expected. However, the Ramachandran plot improvement is an independent metric and its improvement is particularly encouraging. The typical distribution of backbone torsion angles in the Ramachandran plot is brought on by steric hindrance; thus, the improvement of the Ramachandran plot *Z* score is very likely to be the result of using riding H atoms and tight van der Waals restraints in the refinement, which enforce proper steric hindrance. The change in the Ramachandran plot *Z* score strongly depends on resolution (Fig. 3 ▶), with *PDB_REDO* having a stronger impact at resolutions lower than 2.0 Å. This is partly a natural consequence of the original distribution in *Z* scores (there is more room for improvement at lower resolution), but the riding H atoms might also play an important role, since it was (and still is) common practice to not use riding H atoms in lower resolution refinement. This practice is particularly strange considering that riding H atoms add no extra parameters to the refinement, but do add extra restraints or, from an alternative perspective, make already existing van de Waals restraints more effective. This leads to an improvement of the effective data-to-parameter ratio, which is especially important at low resolution.

The *PDB_REDO* pipeline now contains partial model rebuilding and not only re-refinement as in its first implementation. The added value of rebuilding, in combination with a second round of refinement, is made apparent by comparing the distributions in the re-refined and final structure models. In most cases, the rebuilding and extra refinement increases the fraction of models that improve. Interestingly, however, the new steps also increase the fraction of models that become worse according to some of the criteria: this is most evident for *R*_free_ and the number of atomic bumps (an additional 6% of all models became worse according to these criteria).

**Figure 4:**
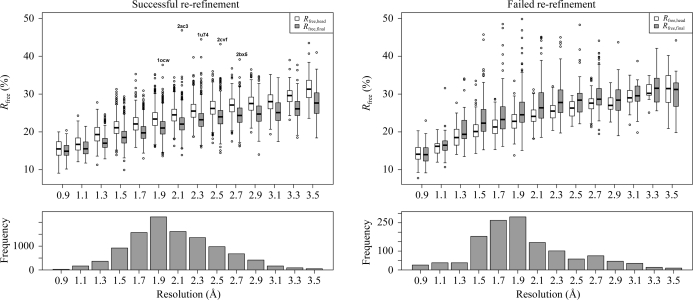
Box-and-whisker plots of *R*_free_ extracted from the PDB header and for the fully optimized *PDB_REDO* models in 0.2 Å resolution bins; the size of each bin is given in the bar chart. The data are divided into two subsets: models for which the initial re-refinement was successful (10 662 models; left) and models for which it failed (1338 models; right). In the case of successful re-refinement *R*_free_ improves over the entire resolution range. The five marked outliers were tested with a new version of *PDB_REDO*: PDB entry 1ocw (James *et al.*, 2003 ▶) was removed from *PDB_REDO* because *R*_head_ could not be reproduced, 2ac3 (Jauch *et al.*, 2005 ▶) and 1u74 (Kang *et al.*, 2004 ▶) were no longer outliers and 2cvf (Akiba *et al.*, 2005 ▶) and 2bx5 (James *et al.*, 2007 ▶) could no longer be re-refined successfully and will be investigated further. If the initial re-refinement fails, *R*_free_ typically increases with many severe outliers.

For the case of *R*_free_, there is however a rather simple explanation. If after the first re-refinement *PDB_REDO* fails to find optimal refinement settings, the model is still rebuilt and refined with automatic geometric restraint weighting. This is likely to explain why the percentage of models with worse *R*_free_ increases in the re-refined and rebuilt set of models. Indeed, for 72% of the models that end up with a worse *R*_free_, we had failed to find a good re-refinement setting in the first place. This is in sharp contrast to the 11% we find in all test cases (incidentally, this is an enormous improvement over the 33% in the first version of *PDB_REDO*). The difference that successful re-refinement makes is made clear in Fig. 4 ▶. In the cases where re-refinement succeeds, all but five structures end up with better free *R* factors at the end. In the cases where re-­refinement fails, even if *R*_free_ still improves on average regardless of resolution, many structures end up having a higher *R*_free_ than that recorded in the header of the starting PDB file. This means that further development of the pipeline should focus on better dealing with this problem or avoiding the problem as much as possible. The latter can be achieved by increasing the number of restraints, *e.g.* by applying jelly-body restraints at higher resolutions or by extending the restraint-weight search space. Optimization of the bulk-solvent mask parameters (the probe size and the shrinkage factor) has recently been implemented and this may also improve the re-refinement results. In 12% of the cases with increased final *R*_free_ the initially calculated *R*_free_ was significantly higher than the *R*_free_ reported in the PDB header [*R*_free,calc_ > 5σ(*R*_free_)_calc_ + *R*_free,head_], while for the total data set this was 5%. Reproducing *R* factors is known to be a challenging problem (Kleywegt *et al.*, 2004 ▶; Afonine *et al.*, 2010 ▶) and many problems can be reduced to a lack of knowledge of the original refinement parameters, *e.g.* about the treatment of bulk solvent. Because the annotation of new PDB entries has improved substantially, we can now adapt *Extractor* to obtain a more detailed description of the original refinement settings. These collectively indicate that part of the problem with worsened *R*_free_ is partly artificial.

**Figure 5:**
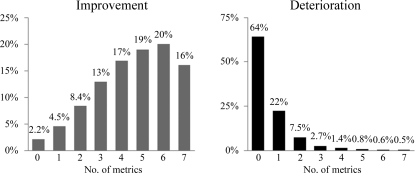
Percentage of structures in the test set as a function of the number of model-quality metrics (see Fig. 2 ▶) that improve (grey; left) or deteriorate (black; right). 85% of the structures improve in three metrics or more, whereas only 6% of the structures deteriorate in three metrics or more.

Deterioration in *R*_free_ does not mean that the model becomes worse in terms of all other quality metrics. In fact, this is very rare and only occurs in 0.5% (58 structures) of the test set (Fig. 4 ▶); deterioration of three or more metrics is still rare and occurs in 6% of the test set. The opposite, improvement of all model-quality metrics, occurs in 16% (1934 structures) of the structures (Fig. 4 ▶), while improvement of three or more quality metrics occurs in 85% of structures in the evaluation set (Fig. 5 ▶).

### 3.2. More constructive validation

During high-throughput testing of *PDB_REDO* on existing PDB entries, more than 800 fixable errors were encountered and were reported back to the PDB. Although mostly trivial annotation problems, these errors can be devastating when structure models are used in automated computational studies. Most issues were resolved at short notice, fixing the problem for all PDB users rather than just for *PDB_REDO*.

### 3.3. Outlook

#### 3.3.1. Ongoing development

The results shown here are encouraging, but also show that there is still ample room for improvement. For instance, the sulfate ion in Fig. 1 ▶(*d*) was distorted, which was traced back to a problem with chiral volume restraints for the S atom. In real-life chemistry the O atoms are equivalent and the sulfur is not chiral. However, the O atoms are different computationally (they have different names), which makes the sulfur chiral during refinement. This means that a swap of any two O atoms in sulfate inverts the chirality of the sulfur. The chiral volume restraints now erroneously push the refinement towards improving the chiral volume, resulting in a distorted molecule. We are currently testing a new tool, called *chiron*, that fixes these computational chirality problems. In the long term, chemical chirality problems (where the atomic coordinates do not match the residue name) should also be fixed by either renaming the compound or rebuilding it. However, this can only be performed when reliable information about the chemical nature of the compound can be obtained automatically, and touches on the issue of constructive validation of ligand entries in the PDB, arguably a more important, but also a significantly more complex, task.

The refinement of a structure model with NCS constraints is available in *REFMAC* and implemented in *PDB_REDO*. Unfortunately, using this option often requires manual intervention because the so-called MTRIX records that describe strict NCS in PDB files frequently have annotation errors. We are working on a decision-making algorithm that can properly deal with such cases.

Our current rebuilding tools can be improved to allow support for noncanonical amino acids such as methylated lysines. Also, more substantial backbone rebuilding, for instance by building missing or poorly defined stretches of many residues, is a target for further improvement of *PDB_REDO*. Adding multiple conformations of side chains and of stretches of main chain presents an additional challenge.

Representation of the optimization results has become much more important now that we are actively changing structure models. We currently use molecular scenes from *YASARA* to highlight changes in the model, but this requires that installation of software and can only show one thing at a time. An approach such as Proteopedia (Hodis *et al.*, 2008 ▶; Prilusky *et al.*, 2011 ▶) could be more flexible and could allow the visualization of results directly in a web browser. Such a tool can allow us to highlight peptides that have been flipped or waters that have been removed.

The current model-quality *Z* scores are robust metrics, but are somewhat less comprehensible by statistically unaware users than, for example, the percentile scores used in *MolProbity* (Chen *et al.*, 2010 ▶); the percentile scores have, besides obvious advantages, some caveats for asymmetric distributions. In addition, the absolute number of bumps that we use in our reports can cause misinterpretation, simply because it does not account for the severity of the bumps. This can lead to misleading results: in absolute terms ten bumps with atomic overlaps of 0.05 Å are worse than a single bump with overlap 0.5 Å even though the former is likely the result of suboptimal restraints and the latter of a fitting error. A new metric for bumps should be developed. Local measures for the fit to the experimental data are not yet included in the *PDB_REDO* pipeline and should be added.

In the near future, the brief validation reports proposed by the PDB Validation Task Force (Read *et al.*, 2012 ▶) are very likely to become the preferred way of presenting *PDB_REDO* results. The relative metrics that are recommended for adoption in this report (and are under development in the PDB) can and should be implemented comparing *PDB_REDO* structures both against average PDB structures but also against average *PDB_REDO* structures.

#### 3.3.2. Using *PDB_REDO*

The *PDB_REDO* pipeline can also be used to refine structures in the process of finalizing a structure in any laboratory. We now regularly employ *PDB_REDO* in our laboratory, usually to optimize near-complete models, but sometimes as early as straight after molecular replacement. While the software is available for download at http://www.cmbi.ru.nl/pdb_redo for use in one’s own laboratory (see, for example, Peng *et al.*, 2010 ▶; Guan *et al.*, 2011 ▶), it is admittedly not straightforward to install and has several system dependencies at the moment. We are also working on a *PDB_REDO* web server that will provide a more user-friendly way to use the *PDB_REDO* pipeline in the near future.

We encourage the usage of *PDB_REDO* prior to structure deposition because it can improve the structure model and its interpretation. It must also be noted that *PDB_REDO* chases a moving target: unlike the PDB models, the *PDB_REDO* data bank models are not static and must all eventually be replaced or supplemented by a new version incorporating new methodological advances.

**Figure 6:**
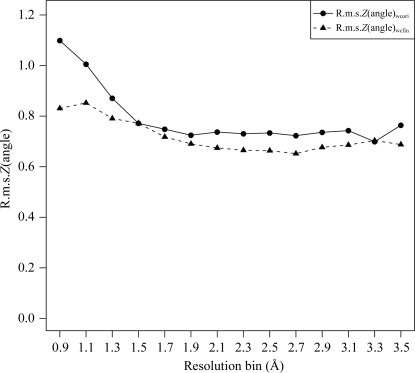
Overall bond-angle deviations from target values expressed as root-mean-square *Z* scores (calculated by *WHAT_CHECK*). Each point is the average of all values in a 0.2 Å resolution bin. Only models with successful initial re-refinement were used. The values in the PDB (wcori; solid line) follow a downward trend to 1.9 Å and then level off; the values after full optimization in *PDB_REDO* (wcfin; dashed line) follow a downward trend to 2.7 Å and then increase. Bond-length deviations (not shown) follow the same trend.

Individual entries in the *PDB_REDO* data bank (http://www.cmbi.ru.nl/pdb_redo) can be used for any structural biology study, *e.g.* for homology modelling (van der Wijst *et al.*, 2010 ▶; Flueck *et al.*, 2011 ▶). Importantly, the collection of models can also be used as a homogeneously treated data set for statistical analysis of structure models. For example, the Ramachandran plot quality (Fig. 3 ▶) or average r.m.s.*Z*(angle) (Fig. 6 ▶) distributions in the *PDB_REDO* differ distinctly from those in the PDB. These distributions can be used to define new refinement targets and new criteria for choosing reliable water molecules or to construct (stricter) validation criteria (Kota *et al.*, 2011 ▶). Further development to alleviate all cases in which some models deteriorate according at least to some criteria will be required before the full potential of *PDB_REDO* can be unleashed for the community.

## 4. Conclusions

The natural and constructive follow-up to structure model validation is to improve the model based on the validation results, as is now common practice by competent X-ray crystallographers. The process of improving the model still requires many decisions to be taken by crystallographers. The *PDB_REDO* pipeline combines refinement and model rebuilding with a decision-making framework that can autonomously optimize structure models. It makes ‘constructive validation’ possible without the need for manual intervention. This is particularly important for structure models in the PDB, which are never updated otherwise. Applying the *PDB_REDO* pipeline to 12 000 random PDB entries showed that the majority of PDB entries can be improved according to commonly accepted quality criteria. This improvement is resolution-dependent: greater improvement occurs at lower resolution. Real-space model rebuilding has substantial added value to re-refinement, especially for improving geometrical targets. The limiting factors to improving models appear to be in finding optimal refinement parameters, but also in extending the scope of rebuilding to larger portions of the main chain and adding multiple conformations. The final hurdles are likely to be uniform and reliable water modelling, and last (but by no means least) the PDB-wide rebuilding of additional macromolecules (nucleic acids and carbohydrates) and the various hetero-compounds (ligands) bound to protein structures.

## Figures and Tables

**Table 1:** Model-quality metrics

Metric	Description
*R*	The standard *R* factor, *R* = 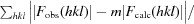 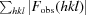 , where *h*, *k* and *l* are the Miller indices of the reflections and *m* is a scale factor. Used with the subscripts head†, calc‡, TLS§, co¶, complex†† and simple††.
*R*_free_	Like *R*, but calculated over a subset of the reflection data (Brünger, 1992 ▶). Subscripts: head†, calc‡, TLS§, co¶, complex††, simple†† and final‡‡.
*R*_ratio_	The expected ratio of *R*_free_/*R* for a converged refinement (Tickle *et al.*, 1998 ▶).
*R*_w_	The weighted *R* factor, *R*_w_ = 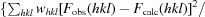 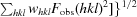 (Hamilton, 1965 ▶), where *w*_*hkl*_ is the the weight for an individual reflection. Subscripts: complex, simple.
*R*_free,w_	The weighted free *R* factor. Subscripts: complex, simple.
σ(*R*_free_)	The estimated standard deviation of *R*_free_: *R*_free_/[2(No. of test-set reflections)]^1/2^. Subscripts: calc (Tickle *et al.*, 2000 ▶).
*R*_free,unb_	The expected *R*_free_ for a converged unbiased refinement (Tickle *et al.*, 1998 ▶): *R*_free,unb_ = *R* × *R*_ratio_. Subscripts: calc.
*Z* score	*Z* = (*x*_model_ − *x*_target_)/σ(*x*), where *x* is a metric such as bond length and σ(*x*) is its standard deviation.
*Z*(*R*_free_)	The *R*_free_*Z* score§§: *Z*(*R*_free_) = (*R*_free,unb_ − *R*_free_)/σ(*R*_free_). Subscripts: calc, complex.
*R*_free,max_	The maximal allowed *R*_free_ value calculated by *picker*.
fit(ρ)	The weighted mean fit of a group of atoms *a* with mean displacement *U* to the map at grid points *x_g_*: WM = 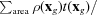 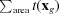 ; *t*(*x*) = 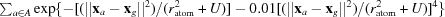 , where *r*_atom_ is the radius of the atom and **x**_*a*_ is the position of the atom (Joosten, Joosten *et al.*, 2011 ▶).
*B*_Wilson_	The Wilson *B* factor.
r.m.s.*Z*(bond)	The root-mean-square *Z* score for *n* bonds with *Z* score *Z_i_*: r.m.s.*Z*(bond) = 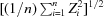 . Subscripts: calc, co, wcori¶¶, wcfin†††.
r.m.s.*Z*(angle)	Like r.m.s.*Z*(bond), but calculated for bond-angle deviations. Subscripts: calc, co, wcori, wcfin.

† Extracted from the header of the input PDB file.

‡ Calculated by *REFMAC* before refinement.

§ Calculated during TLS refinement in *REFMAC* directly after resetting the *B* factors.

¶ Used as a cutoff value for *picker*.

†† Complex refers to the model with the most (*B*-factor-related) parameters and simple to the model with fewest.

‡‡ Calculated by *REFMAC* after the final refinement.

§§ The terms are swapped to compensate for the ‘lower-is-better’ nature of *R*_free_.

¶¶ Calculated by *WHAT_CHECK* for the input PDB file.

††† Calculated by *WHAT_CHECK* for the final model.

**Table 2:** Programs in the *PDB_REDO* pipeline

Program	Software suite	Application in *PDB_REDO*
*Stripper*	*PDB_REDO*	Removes unwanted atoms and edits LINK records in PDB files
*Cif*2*cif*	*PDB_REDO*	Checks and standardizes reflection data in mmCIF files
*Extractor*	*PDB_REDO*	Extracts the description of the structure model and refinement from a PDB file
*Fitr*	*PDB_REDO*	Compares *R* factors
*Chiron*	*PDB_REDO*	Fixes chirality errors
*Bselect*	*PDB_REDO*	Selects *B*-factor model complexity
*Picker*	*PDB_REDO*	Selects the best refinement from a set
*Centrifuge*	*PDB_REDO*	Removes waters
*SideAide*	*PDB_REDO*	Real-space rebuilds side chains and add missing ones
*pepflip*	*PDB_REDO*	Flips peptide planes
*What_todo*	*PDB_REDO*	Parses *WHAT_CHECK* reports for *SideAide*
*REFMAC*	*CCP*4	Performs reciprocal-space refinement
*TLSANL*	*CCP*4	Checks TLS-group definitions and converts total *B* factors to residuals
*CIF*2*MTZ*	*CCP*4	Converts reflection data from mmCIF to MTZ format
*MTZ*2*VARIOUS*	*CCP*4	Converts reflection data from MTZ to mmCIF format
*CTRUNCATE*	*CCP*4	Converts reflection intensities to amplitudes
*MTZUTILS*	*CC*P4	Manipulates MTZ files
*CAD*	*CCP*4	Merges MTZ files
*UNIQUE*	*CCP*4	Creates all possible reflections given unit-cell parameters and resolution
*FREERFLAG*	*CCP*4	Creates and completes *R*_free_ set
*SFCHECK*	*CCP*4	Calculates completeness, twinning fraction and *B*_Wilson_
*DSSP*	—	Assigns secondary structure
*Umfconverter*	*PDB-care*	Validates carbohydrates in structure model
*WHAT_CHECK*	*WHAT IF*	Validates the structure model
*Pdbout*2*html*	*WHAT IF*	Converts *WHAT_CHECK* validation reports to html
*YASARA Structure*	*YASARA*	Creates scenes for result visualization

**Table 3:** Structure-model categories

	Cutoff values	
Category	Reflections per atom†	Data resolution
xlow	<1.0 reflections per atom	Resolution ≥ 5.00 Å
vlow	1.0 ≤ reflections per atom < 2.5	3.50 Å ≤ resolution < 5.00 Å
low	NA†	2.80 Å ≤ resolution < 3.50 Å
medium	NA†	1.70 Å ≤ resolution < 2.80 Å
high	NA†	1.20 Å ≤ resolution < 1.70 Å
atomic	NA†	Resolution < 1.20 Å

† Reflections per atom takes precedence over data resolution.

‡ In these categories only resolution cutoffs are used.

**Table 4:** Whole data-set averages for model-quality metrics

Metric	PDB entry	Re-refined model	Final model
*R* (%)	19.8†	18.3	18.4
*R*_free_ (%)	24.0†	22.0	22.2
Ramachandran plot‡	−1.30	−0.66	−0.61
Side-chain rotamers‡	−1.21	−0.69	−0.24
Coarse packing‡	−0.24	−0.16	−0.12
Fine packing‡	−0.97	−0.85	−0.70
No. of atomic bumps	108	78	82
No. of unsatisfied hydrogen-bond donors/acceptors	43	37	37

† Values extracted from the PDB header.

‡ Model-normality *Z* scores from *WHAT_CHECK* with respect to a test set of 500+ high-resolution structure models. Higher values are better.
